# The Genetics of Extreme Longevity: Lessons from the New England Centenarian Study

**DOI:** 10.3389/fgene.2012.00277

**Published:** 2012-11-30

**Authors:** Paola Sebastiani, Thomas T. Perls

**Affiliations:** ^1^Department of Biostatistics, Boston University School of Public HealthBoston, MA, USA; ^2^Section of Geriatric, Department of Medicine, Boston University School of Medicine and Boston Medical CenterBoston, MA, USA

**Keywords:** centenarians, genetic of longevity, heritability of longevity, compression of morbidity, genetic variation

## Abstract

The New England Centenarian Study (NECS) was founded in 1994 as a longitudinal study of centenarians to determine if centenarians could be a model of healthy human aging. Over time, the NECS along with other centenarian studies have demonstrated that the majority of centenarians markedly delay high mortality risk-associated diseases toward the ends of their lives, but many centenarians have a history of enduring more chronic age-related diseases for many years, women more so than men. However, the majority of centenarians seem to deal with these chronic diseases more effectively, not experiencing disability until well into their nineties. Unlike most centenarians who are less than 101 years old, people who live to the most extreme ages, e.g., 107+ years, are generally living proof of the compression of morbidity hypothesis. That is, they compress morbidity and disability to the very ends of their lives. Various studies have also demonstrated a strong familial component to extreme longevity and now evidence particularly from the NECS is revealing an increasingly important genetic component to survival to older and older ages beyond 100 years. It appears to us that this genetic component consists of many genetic modifiers each with modest effects, but as a group they can have a strong influence.

## The New England Centenarian Study and Exceptional Longevity

The New England Centenarian Study (NECS)^1^ was founded in 1994 as a population-based study of all centenarians living within eight towns in the Boston area with the goal of better understanding the bio-psycho-social characteristics of centenarians and their family members and discovering determinants of exceptional longevity and healthy aging (Perls et al., 1999). The study soon expanded enrollment to include siblings of centenarians and their offspring from throughout North America, and since 2008 there has been a particular effort to locate and recruit subjects 105 years old and older (what Nobu Hirose of the Japanese Centenarian Study has termed “semi-supercentenarians”). In addition to recruiting long lived individuals, the NECS has also recruited younger referent subjects from families lacking longevity as well as spouses of centenarians’ offspring. Between 1994 and 2012, the study has enrolled more than 1,800 centenarians and 123 supercentenarians (age 110+ years), more than 600 centenarian offspring and 437 controls. The majority of the NECS centenarians were born between 1880 and 1910 and reached a median survival of 103 years, thus surviving 30–40 years past the median survival of their birth year cohort. Birth certificates were available for only about 30% of the centenarians and therefore US census data from the early 1900s and other techniques were used for validating date of birth (Young et al., 2010; Andersen et al., 2012). Typically, 99% of age claims 115 years and older are false, and therefore in the case of supercentenarians, the NECS takes extra steps to prove a person’s age including family reconstitution and collecting multiple forms of proof that all must be consistent with one another (Young et al., 2010). Demographic, health, and family history data, as well as physical and cognitive function data are collected at least once for the majority of study subjects and are updated annually for living subjects. DNA samples have also been collected on the majority of subjects.

## What is the Cut Off Age for Exceptional Survival?

Birth cohort life tables provide an indication of the exceptional survival of centenarians (Bell and Miller, 2005). Based on the U.S. Social Security Administration birth cohort life table,^2^ the median survival for males born between 1896 and 1905 was 63 and 72 years for females and 1% of males and 5% of females lived past the age of 95 years. 0.1% of males and 1% of females lived past the age of 100 (Bell and Miller, 2005). The frequencies of survivors past the age of 100 decrease by a factor of approximately one half for each additional year of life after 100. Their life span represents a phenomenon of extreme survival that is very rare in the population, and much more extreme than the human longevity examined for example in the Leiden Study of Longevity (Deelen et al., 2011), in which subjects reached an average survival of 94 years, or the CHARGE consortium (mean age 81 years; Walter et al., 2011).

Though still rare, the prevalence of people at age 100 years is growing. When the NECS began in 1994, the estimated prevalence in the US and other developed nations was one centenarian per 10,000. Now, in 2012, the prevalence has doubled to 1 per 5,000. One explanation for such growth is that at the turn of the last century, marked improvements were taking place in public health, particularly ones which impacted upon neonatal and maternal mortality. In 1900, infant mortality was 10–30% for the first year of life depending upon the area of the country and 6–9% of women died due to complications of childbirth.^3^ Cleaner water supplies, plumbing, milk pasteurization, marked socioeconomic improvements, vaccines, and a major increase in the mean years of education led to major improvements in survival. Then in the 1930s and 1940s, the introduction of antibiotics (sulfa and penicillin), additional vaccines, safe blood transfusion, and of course many other medical and non-medical advancements led to marked further improvements in infant survival and also survival in adulthood. Figure 2 in Armstrong et al. (1999), illustrates the dramatic decline in mortality due to infectious diseases in the United States from 1900 to 1960. Now, in the last 20 or so years, there has also been a marked effect upon survival to 100 due to reduced mortality rates amongst the geriatrics population (Vaupel et al., 1998).

The result is that with these improvements in our environment, many more people that might have otherwise died during infancy have the opportunity to take advantage of their longevity potential and live to much older age. For example, according to James Evans of the UK’s Department of Work and Pensions, a 20-year-old today has a three times greater chance of living to age 100 than when their grandparents were 20 (Evans, 2011). That work produced Table 1 indicating the probabilities of men and women from different birth cohorts living to 100.

**Table 1:** **Chance of living to age 100 according to birth year and sex (directly from with permission)**.

Year of birth	Male (%)	Female (%)
1931	2.5	5.1
1961	10.5	16.2
1991	19.2	26.4

Kaare Christensen and coauthors provide an even more optimistic picture predicting that 50% of French girls born in 2010 will live to 100 (Christensen et al., 2009). Certainly, a phenotype achieved by 50% of the population can not be considered exceptional. However, such projections assume that these large proportions of the population have the biological wherewithal to survive to 100+ years if given the appropriately facilitative environment and this really is not known.

As discussed below there is a growing body of evidence indicating that an increasingly greater positive genetic influence is necessary for survival to age 100 and older ages. Presumably, the vanishing small proportion of people that is able to reach the ages in the extreme tail of the population is due to the increasing rarity of genetic and environmental factor combinations that improve the odds of such rare survival. Supporting this hypothesis is the observation that while the percentage of the population made up of 100 year olds over the past 10 years might still be climbing, the rate of people living to ages 112+ has remained flat.^4^ The reason for this could be that specific and very rare genetic signatures are necessary to achieve these ages. For younger ages, for example 105–109 years, the necessary genetic/environmental signatures may be less rare and not all people who have the potential to achieve these ages have yet done so, thus we see a continued growth in their prevalence rate. Once we have a better idea of who is predisposed and by how much, we will be better able to understand what is exceptional longevity versus average and perhaps even below average longevity. Complicating matters however is that signatures are likely different according to some ethnicities and some specific environmental exposures and therefore definitions of exceptionality will vary within these contexts. This complex model of many common and rare genetic variants with modest effects collectively having an increasingly strong effect on survival to older and older ages speaks to the importance of centenarian studies from around the world working closely with one another to effectively compare and contrast their findings.

## Exceptional Health Span and the Compression of Morbidity Hypothesis

Ever since Jim Fries proposed his compression of morbidity hypothesis, published in the New England Journal of Medicine in 1980, most researchers in Gerontology believed that in order to survive to 100 years, one necessarily had to markedly delay both morbidity and disability toward the end of their life. Then, in 2002 Jesse Evert, collected the data and published findings that less than 20% of centenarians had escaped major age-related diseases by the time they reached the age of 100 and approximately 45% developed at least one of these diseases before the age of 65 (Evert et al., 2003). However, consistent with at least part of Fries’ hypothesis, we also previously noted that despite the presence of diseases, approximately 90% of centenarians delayed disability until the mean age of 93 years indicating perhaps greater functional reserve that enabled these individuals to remain independent for a long time despite the presence of diseases associated with high risks of disability and mortality (Hitt et al., 1999).

In the past 7 years, as the NECS emphasized the enrollment of even older subjects, those 105+ years, we noted much later ages of onset of age-related diseases. At this point we surmised that Dr. Fries had underestimated the practical limit of human life span (e.g., 100 years) and we simply had not looked at old enough ages to test his hypothesis that age-related diseases and disability were compressed to shorter and shorter periods of time as subjects approached the limit of life span. Once 100 of our subjects had reached 110+ years, we analyzed our longitudinal data again and indeed found that at progressively older and older ages of survival beyond 100 years, there is a progressive compression of disability and morbidity such that by the survival age of 110 years, subjects had compressed age-related diseases into the last 5.2% of their extremely long lives (compared to 17.9% for controls, 9.4% for subjects age 100–104 years, and 8.9% for those age 105–109 years; Andersen et al., 2012). Furthermore, at these most extreme ages, subjects became much more alike in terms of the types and ages of onset of age-related diseases. This increased homogeneity at oldest ages and our genetic findings to-date hold great promise for many more findings regarding factors that facilitate slower aging and the delay or escape of age-related diseases.

## Evidence for a Strong Familiality of Exceptional Longevity

Twin studies have shown that only 20–30% of the overall variability of living to the mid 80s is attributable to genetic variation (Herskind et al., 1996), and this result is unfortunately and erroneously used to indicate the heritability of exceptional longevity. Work by the Adventist Health Study indicates that with optimal health related behaviors (e.g., no tobacco or alcohol use, regular exercise, vegetarianism, effective management of stress) people should generally be able to achieve an average life expectancy of about 86 years (Fraser and Shavlik, 2001). This suggests that the average genome, in combination with optimal health behaviors, facilitates an average life span of the late eighties and it would make sense that the vast majority of why one lives to their sixties or seventies versus these later octogenarian years would be explained by health habit choices. As indicated above though, the odds of living to the mid-eighties are many folds more common than living to 100 years or older and it is likely that these twin studies cannot inform us about the heritability of living to 100.

**Figure 1:**
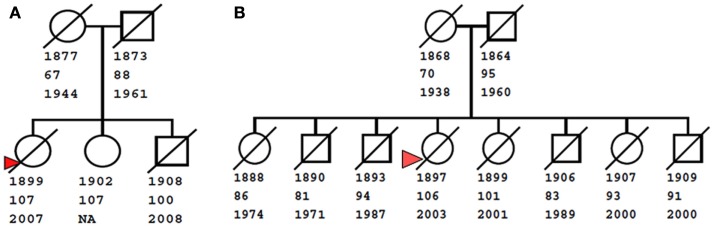
**Examples of familial clusters of exceptional longevity**. The small pedigree on the left **(A)** shows a sibship of three centenarians, with youngest age at death of 100 years. The pedigree on the right **(B)** shows a larger sibship of eight, with two centenarians (ages at death 101 and 106 years), three nonagenarians (ages 91, 93, and 94), and three siblings who live past the age of 80. Squares and circles represent males and females, diagonal bars represent deceased subjects. Numbers below nodes are birth years, last age at contact, and death year. For living subjects, the death year is not available (NA). Red triangles denote probands enrolled in the New England Centenarian Study.

The NECS has enrolled several 100 families with centenarian siblings and these data have provided increasingly stronger evidence that exceptional longevity clusters in families (Perls et al., 1998, 2000). As an example, Figure 1 shows two centenarians and their siblings enrolled in the NECS who reached remarkable ages. The rarity of such families with clusters of exceptional longevity was discussed in (Perls et al., 2000). The exceptionality of familial longevity in a larger number of NECS sibships relative to Framingham Heart Study sibships was also described in (Sebastiani et al., 2009) using an objective measure of familial longevity. Less than 1% of all the families in the FHS would meet the exceptionality of the sibships enrolled in the NECS. Additionally, the NECS has also shown that siblings of centenarians had between 8 and 17 times greater chances of living past 100 years compared to individuals from the same birth year cohort (Perls et al., 2002).

The sex-specific sibling relative risk and the prevalence of centenarians estimated as 1 centenarian every about 5,000 individuals in the US population can be used to estimate the heritability of the liability of living past 100 using formulas in (Wray et al., 2010). Using the online calculator http://gump.qimr.edu.au/genroc/ the heritability of liability to longevity ranges between 0.33 (females) and 0.48 (males). Estimates of relative risk of exceptional longevity for siblings of semi-supercentenarians and supercentenarians are needed to have a better understanding of the heritability of living to extreme ages even greater than 100.

While variability in average life span can be explained by environmental factors and genetics, exceptional longevity seems to be more the resultant of genetics rather than environment. Recently, the Ashkenazi Jewish Centenarian Study has shown that centenarians in their study do not differ from population controls in major risk factors such as increased BMI, drinking, or smoking (Rajpathak et al., 2011). Although lifetime exposures in centenarians are difficult to measure in a reliable way, this analysis suggests that environmental factors have little contribution to extreme longevity, so that most of the heritability of the trait is likely to have a genetic basis.

## Genetic Influence upon Survival to Very Old Ages

**Figure 2:**
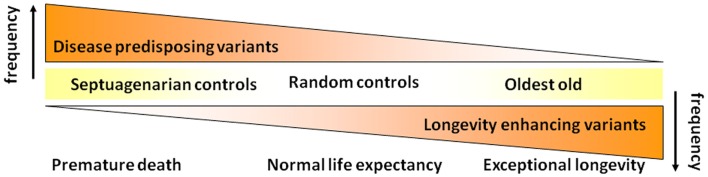
**Initial hypothesis on the genetic basis of exceptional longevity**.

Per the phenomenon of demographic selection (Vaupel et al., 1979; Carey and Judge, 2001), we initially hypothesized that centenarians necessarily lack genetic variants associated with premature mortality and perhaps additionally also have genetic variants associated with slower aging and reduced risk for age-related diseases and subsequent mortality, so-called “longevity enabling genes” (see Figure 2).

Since there are many different phenotypic presentations of survival to 100 years we also expected that many genetic variants contributed to a complex genetic model for exceptional longevity. Complicating matters, environmental factors which can be deleterious (e.g., tobacco use is associated with specific cancers in some people and not others) and others which can be health-promoting (e.g., daily aspirin use), will effect survival risk-associated with a person’s genetic profile.

To be able to discover the complex genetic basis of exceptional longevity and dissect the trait into sub-phenotypes that represent different patterns of exceptional survival, we conducted a genome-wide association study of exceptional longevity with 801 unrelated centenarians from the NECS (median age at death 104 years) and 914 genetically matched controls from the Illumina control repository and the NECS. The initial version of the results in Science Express in July 2010 contained errors and we retracted it (Sebastiani et al., 2011). We published the corrected version in (Sebastiani et al., 2012b) after extensive cleaning of the data and independent validation of the genotype data. Analysis of approximately 240,000 single nucleotide polymorphisms (SNP) showed that only one SNP in TOMM40/APOE (rs2075650) reached irrefutable genome-wide significance, while a large number of SNPs were significantly associated with exceptional longevity but did not pass corrections for multiple comparisons (*p*-value for association between 10^−2^ and 10^−7^). This result was consistent with other studies of longevity that failed to identify genome-wide significant associations beyond APOE but detected many associated SNPs with more moderate levels of significance (Newman et al., 2010; Deelen et al., 2011; Walter et al., 2011). In addition to traditional one-SNP-at-a-time analysis we introduced a new method to capture the simultaneous effect of many genetic variants that individually have minor to modest effects, but as a group assert a substantial influence upon survival to extreme old age. The method comprised essentially three steps:

1. The first step builds a set of mathematical models that can be used together to distinguish centenarians from individuals selected from the general population using only genetic data.
2. The second step uses the set of models to generate a genetic risk profile of exceptional longevity for each study subject. These genetic risk profiles of centenarians can be analyzed using cluster analysis that essentially groups centenarians based on different patterns of genetic risk. We termed the average profiles associated with these clusters as “genetic signatures” of exceptional longevity and they represent combinations of genetic variants that produce a similar chance or probability for exceptional longevity.
3. The third step correlates these genetic signatures with different phenotypic paths to exceptional longevity, to begin to understand what genetic variants are associated with different patterns of exceptional longevity.

For step 1, we used a class of Bayesian classification models that are suitable to analyze case-control studies and developed a forward search algorithm to build nested models with increasing numbers of SNPs and a stopping rule based on sensitivity and specificity of the classification models. To derive the models, all approximately 240,000 SNPs were ranked according to their posterior probability of association with exceptional longevity, and SNPs in strong linkage disequilibrium were pruned out (Sebastiani et al., 2012b). Then beginning with the SNP demonstrating the strongest probability, a set of nested Bayesian classification models was built by adding one SNP at a time from the sorted list of SNPs, and the sensitivity (centenarians predicted as centenarians) and specificity (controls predicted as controls) was assessed. SNPs were no longer added when both the specificity and sensitivity did not increase significantly. These types of Bayesian models can be mathematically related to logistic models with a genetic risk score (Sebastiani et al., 2012c), but they can be adapted more easily to include multiple traits and additional covariates (Hartley et al., 2012).

This algorithm identified 281 SNPs in 130 genes and intragenic regions that include well known aging and age-related disease genes, but also novel genes. The set of models was used together to distinguish between centenarians and controls using an average (ensemble) of the predictions of each single model. It is well known that an ensemble of prediction models usually outperform single “best” models because it is more robust to inclusion of false positive SNPs (Rokach, 2010).

To validate this set of models, we tested their joint accuracy to distinguish between centenarians and controls in independent sets of centenarians and controls reaching 60% specificity and a sensitivity between 58 and 85% (the older the centenarians the greater the sensitivity). The sensitivity of the set of models to distinguish controls from nonagenarians and older was low (58%), but was higher to distinguish controls from older centenarians. This result is consistent with an increasing genetic contribution to reach older and older ages beyond 100 years. The lower specificity could be due to the fact that longevity variants are also present in the controls and that other non-genetic factors may be needed to for individuals to survive to very extreme ages.

**Figure 3:**
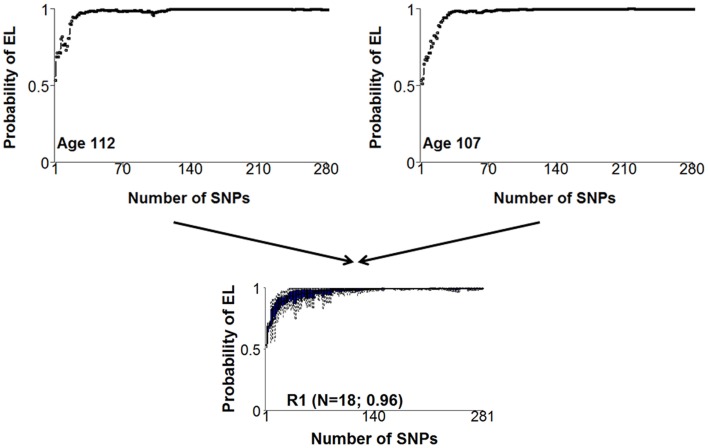
**Examples of two similar genetic risk profiles that are highly predictive of exceptional longevity and a cluster of profiles that include the two profiles**. The *x*-axis displays the 281 SNPs, sorted by the significance of the association with exceptional longevity. The *y*-axis represents the posterior probability of exceptional longevity given the nested sets of SNPs. The two genetic risk profiles in the top panel represent the pattern of risk for varying combinations of genotypes of the 281 SNPs that we found associated with exceptional longevity. The two plots are not exactly the same, meaning that the two subjects have some different alleles of the 281 SNPs. However, the similar pattern of risk means that they essentially have the same genetic basis for exceptional longevity and were assigned to the same cluster displayed in the bottom panel.

In step 2, the set of 281 SNPs and models were used to first summarize the prediction of the set of nested models in the form of genetic risk profiles. The display of genetic risk profiles provides information about the enrichment of longevity variants of an individual because subjects with similar genetic profiles share most of the longevity SNPs and therefore a similar risk for longevity (See Figure 3). By cluster analysis of the genetic risk profiles, centenarians with similar genetic risk profiles can then be grouped to generate what we called genetic signatures of exceptional longevity. We used a Bayesian model-based cluster analysis that is described in (Ramoni et al., 2002) to group centenarians with similar genetic risk profiles. This approach essentially clusters centenarians with genetic risk profiles that follow the same probability distribution. Full details are in (Sebastiani et al., 2012b). We further found that different genetic signatures correlate with significantly different life spans and significantly different ages of onset of major age-related diseases such as dementia, cancer, and cardiovascular disease. Note that while a genetic risk profile informs about the enrichment of risk variants that an individual carries, genetic signatures represent the prevalent genetic risk profiles that lead to different risks. Therefore, genetic signatures can be used for dissection of a complex trait, patient stratification, experimental design, and understanding the mechanism that links genotype to phenotype.

## The Role of Disease Alleles and Longevity

One of our hypotheses about the genetic make-up of exceptional longevity was that a relative lack of disease-associated variants could in part explain centenarians’ survival advantage. To test this hypothesis we computed the rate of SNP alleles that were associated with a variety of diseases and traits in several GWASs from the catalog of published genome-wide association studies^5^ (Hindorff et al., 2009) and the Human Gene Mutation Database (HGMD; Stenson et al., 2009). We found 1,214 of the 62,339 disease-associated SNPs in the GWAS of exceptional longevity and we did not observe a significant difference in the rate of disease-associated variants carried by centenarians and population controls. The analysis was conducted by stratifying the rates by disease type and in none of the 14 groups of disease that we analyzed did we note a difference between centenarians and a large number of controls from the NECS and other GWAS. This result agreed with recent findings from the Leiden Longevity and Leiden 85+ Studies that in their nonagenarian sample, the rate of disease-associated variants for a select group of age-related diseases was the same as in the general population (Beekman et al., 2010).

Although this analysis is incomplete because the genotype data did not include most of the known disease variants and the Illumina controls are likely healthy, we noted an equivalent result in the whole genomes of two supercentenarians (Sebastiani et al., 2012a). These two genomes included only 1% of mutations from the HGMD and approximately 50% of the mutations that were linked to common diseases in genome-wide association studies. These disease mutations included known SNPs linked to age-related diseases such as Alzheimer’s and ALS, cancer and cardiovascular disease. Compared to 11 other whole genome sequences, the two supercentenarian genomes carried a rate of disease variants comparable to the Venter and Watson genomes and even higher rates compared to Caucasians from the 1,000 Genomes project.

**Figure 4:**
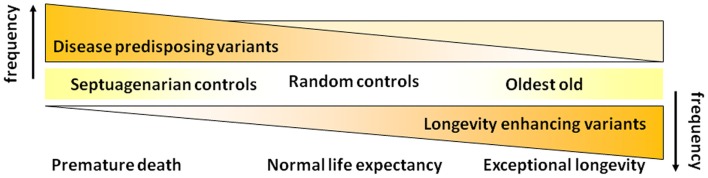
**Revised hypothesis for the prevalences of disease and longevity (or protective) associated variants with increasing age at very old ages**.

Realizing that disease-associated variants are not necessarily weeded out of the population as people survive to the most extreme ages, we have subsequently revised the hypothesis that we began our studies with (Figure 2). As shown in Figure 4, we now hypothesize that with increasing age there is not so much a decline in the prevalence of disease-associated genetic variants but rather there is a selection for longevity-associated variants which not only can counter the deleterious effects of genetic and environmental factors but also afford protection against basic mechanisms of aging, slow the rate of aging and delay the onset of age-related diseases, and syndromes.

## Noticeable Genes Linked to Exceptional Longevity and Follow Ups

The 281 SNPs that we found predictive of exceptional longevity include SNPs in genes associated with age-related diseases such as Alzheimer’s, dementia, and cardiovascular disease. Although the SNP rs2075650 in TOMM40/APOE reached genome-wide significance and the AA genotype is associated with increased odds for exceptional longevity, its effect was limited and we showed that this SNP explains only 1% of the predictive accuracy of the joint set of 281 SNPs (Sebastiani et al., 2012b). The GG genotype of this SNP is linked to the ε4 allele of the *APOE* gene and while carriers of this genotype are clearly predisposed to early mortality, carriers of the AA genotype seem to have a small survival advantage compared to AG carriers (Schupf et al., 2012). We are currently conducting replication studies of these 281 SNPs to identify the most robust longevity variants to be carried forward in functional studies. The centenarians’ genomes that we published in (Sebastiani et al., 2012a) suggest that most of the SNPs in the list of 281 are located closer to coding SNPs compared to randomly chosen SNPs. For example, the two genomes included more than 50% of the longevity variants in genes and approximately 20% of these mutations were within 10 kb from coding mutations. These data helped us identify new coding mutations for example in the progeria genes LMNA and WRN which is particularly interesting since these findings suggest that different variations of these genes may lead to either extreme premature aging or extreme longevity. Details of the specific mutations are in (Sebastiani et al., 2012a).

## Conclusion

People surviving to 100 years and older generally delay the onset of disability well into their nineties. For those who survive to the most extreme ages, for example beyond 105 years, we have observed a progressive compression of morbidity as well. These oldest of the old individuals also appear to be more phenotypically homogeneous compared to people surviving to just 100 years. Numerous genome-wide association studies of centenarians have yielded only very few statistically significant findings. The most notable and consistent of these is a variant of apolipoprotein E which has been shown in candidate gene studies to be the variant APOE ε4 which is both rare and deleterious in centenarian studies of various ethnicities. Interestingly though, for some ethnicities, the frequency of this allele can be substantially greater. Analyses of sibships that cluster for exceptional longevity and the increasing but rare phenotypic homogeneity of the most extreme old still suggest a strong genetic component. Thus, the NECS and other studies hypothesize that exceptional longevity is a complex trait influenced by multiple genetic variants that individually have modest effects, but as a group can exert a strong effect. Using a Bayesian analytic approach to construct a genetic model that predicts exceptional longevity, we have found that with older and older age beyond 100 years, this genetic influence appears to get stronger and stronger. While the APOE ε4 allele has garnered much attention for its negative association with exceptional longevity, we found that its contribution to distinguishing between centenarians and healthy controls is minimal (Sebastiani et al., 2012b). Also counter to the conventional wisdom, now several studies have shown that centenarians have many of the disease-associated variants found in the general population thus suggesting an important role for protective variants that counter the effects of these deleterious variants as well as possibly slow the rate of aging and the decrease the risk for age-related diseases that contribute to premature mortality. Genetic models such as these may be used to discover new target genes and pathways related to aging and longevity.

## Conflict of Interest Statement

The authors declare that the research was conducted in the absence of any commercial or financial relationships that could be construed as a potential conflict of interest.

## Footnotes

^1^http://www.bumc.bu.edu/centenarian

^2^http://www.ssa.gov/oact/NOTES/as120/LifeTables_Tbl_7_1900.html

^3^http://www.cdc.gov/mmwr/preview/mmwrhtml/mm4838a2.htm

^4^http://www.mapfre.com/fundacion/html/revistas/gerencia/n111/docs/Estudio1En.pdf

^5^http://www.genome.gov/26525384
